# Impact of *Cordyceps sinensis* on coronary computed tomography angiography image quality and renal function in a beagle model of renal impairment

**DOI:** 10.3389/fphar.2025.1538916

**Published:** 2025-01-29

**Authors:** Peiji Song, Kun Li, Xiaodie Xu, Guifeng Zhang, Zengkun Wang, Linbing Sun, Zekai Zhao, Ting Li, Ximing Wang, Zhangyong Xia

**Affiliations:** ^1^ First Clinical Medical College, Shandong University of Traditional Chinese Medicine, Jinan, Shandong, China; ^2^ Department of Radiology, Central Hospital Affiliated to Shandong First Medical University, Jinan, Shandong, China; ^3^ Department of Neurology, Liaocheng Hospital Affiliated to Shandong First Medical University and Shandong Academy of Medical Sciences, Liaocheng, Shandong, China; ^4^ Department of Radiology, Shandong Provincial Hospital Affiliated to Shandong First Medical University, Jinan, Shandong, China; ^5^ Department of Neurology, The Second People’s Hospital of Liaocheng, Liaocheng, Shandong, China; ^6^ Department of Neurology, Liaocheng People’s Hospital, Liaocheng, Shandong, China; ^7^ State Key Laboratory of Dampness Syndrome of Chinese Medicine, Shandong Sub-Centre, Liaocheng, Shandong, China

**Keywords:** *Cordyceps sinensis*, low dose CM, coronary CT angiography, Beagle dogs, renal function, functional magnetic resonance imaging

## Abstract

**Objective:**

This study aims to investigate the protective effects of *Cordyceps sinensis* against renal injury induced by low-dose contrast medium (CM) in coronary computed tomography angiography (CCTA) imaging, and to evaluate its efficacy using functional magnetic resonance imaging (fMRI).

**Methods:**

Twenty Beagle dogs with induced renal insufficiency were enrolled in the study and randomly assigned to one of four groups (n = 5 per group). Group A received *Cordyceps sinensis* for 1 week prior to undergoing heart rate-dependent personalized CM CCTA scanning; Group B received *Cordyceps sinensis* for 1 week followed by conventional dose CM CCTA scanning; Group C did not receive *Cordyceps sinensis* but underwent HR-dependent CM CCTA scanning; and Group D did not receive *Cordyceps sinensis* but underwent conventional dose CM CCTA scanning. Renal function was assessed using MRI before and after the intervention, with IVIM (Intravoxel Incoherent Motion) and BOLD (Blood Oxygen Level Dependent) imaging of the kidneys. Key parameters, including the pure diffusion coefficient (D), pseudo-diffusion coefficient (D*), perfusion fraction (f), and R2*values, were quantified. Laboratory renal function markers were measured multiple times before and after the intervention, and their correlation with fMRI parameters was analyzed.

**Results:**

CCTA imaging revealed that the CT values of the major coronary artery branches in all groups met the international diagnostic criteria for coronary arteries. No statistically significant differences in image quality were observed among the four groups (P > 0.05). In Groups A and D, significant changes were observed in renal function parameters, as well as in D, D*, f, and R2* values, both pre- and post-CCTA (P < 0.05). However, Groups B and C exhibited no significant changes pre- and post-CCTA (P > 0.05). A significant correlation was found between MRI parameters and laboratory renal function markers, with excellent inter- and intra-observer reproducibility, and high repeatability in the measurements.

**Conclusion:**

HR-dependent personalized CM CCTA imaging did not compromise image quality. Administration of *Cordyceps sinensis* demonstrated a potential protective effect on renal function. The combination of IVIM and BOLD functional MRI offers a reliable, non-invasive approach to assess the protective effects of *Cordyceps sinensis* on renal injury induced by low-dose CCTA in Beagle dogs.

## 1 Introduction

Coronary atherosclerotic heart disease is one of the leading causes of death worldwide. Coronary computed tomography angiography (CCTA) has become a key non-invasive diagnostic tool widely used in clinical practice for assessing the hemodynamic significance of coronary artery disease (Koo et al., 2024). However, in patients with renal insufficiency, the use of CM poses a risk of inducing renal injury. Therefore, it is crucial to identify imaging techniques that can maintain diagnostic image quality while minimizing renal function impairment (Davenport et al., 2020; Weisbord and Palevsky, 2011; Mousavi Gazafroudi et al., 2019; Chandiramani et al., 2020; Do, 2017; Fähling et al., 2017; Pistolesi et al., 2018; Lee et al., 2019; Mirhosseni et al., 2019; Kusirisin et al., 2020; Li and Pan, 2022).

The diagnosis and staging of chronic kidney disease (CKD) typically rely on the assessment of renal structural changes and functional markers. In recent years, magnetic resonance imaging (MRI) has gained significant attention in kidney disease research due to its non-invasive nature, ease of operation, and good reproducibility (Liang et al., 2016; Zhang and Lee, 2020; Wang et al., 2021; Zhou et al., 2023; Tao et al., 2024a; Zhu et al., 2024). Functional MRI (fMRI) not only allows for visualization of anatomical changes in the kidneys but also enables non-invasive and quantitative monitoring of renal function and pathophysiological alterations (Liang et al., 2016; Zhang and Lee, 2020; Wang et al., 2021; Zhou et al., 2023; Tao et al., 2024a; Mao et al., 2023). Among the various fMRI techniques, Intravoxel Incoherent Motion (IVIM) and Blood Oxygen Level Dependent (BOLD) imaging have been widely applied in clinical settings and animal models to monitor kidney function in CKD patients (Li et al., 2014; Yang et al., 2019; Wang et al., 2019a; Wang et al., 2019b; Li et al., 2021; Ren et al., 2023). IVIM imaging provides parameters such as the true diffusion coefficient (D), pseudo-diffusion coefficient (D*), and perfusion fraction (f), which are used to quantitatively assess blood flow changes in solid organs (Mao et al., 2018; Zhang et al., 2018; Cheng et al., 2024; Zhou et al., 2024). BOLD imaging, on the other hand, evaluates renal oxygenation status by measuring the apparent transverse relaxation rate (R2* value = 1/T2). The R2* value is negatively correlated with the oxygen partial pressure in tissues and positively correlated with the level of deoxygenated hemoglobin (Wang et al., 2022).

*Cordyceps sinensis*, a traditional Chinese medicinal herb, has been extensively studied in recent years for its therapeutic potential in various diseases, particularly for its renal protective properties. *Cordyceps sinensis* plays a therapeutic role in CKD by regulating immune response, oxidative stress and inflammatory response through multiple targets, such as neuroactive ligand-receptor interaction, insulin resistance, chemical oncogenic receptor activation, cAMP signaling pathway and other pathways (Tao et al., 2024b; Zhang et al., 2022; Liu et al., 2022). This study aims to investigate the application of low-dose CM CCTA in a Beagle dog model of renal insufficiency, assess the renal protective effects of *Cordyceps sinensis*, and evaluate these effects using functional MRI (fMRI).

## 2 Materials and methods

### 2.1 Animals

The animal study was reviewed and approved by Ethics Review Committee of Shandong First Medical University [NO.W202403040242]. Prior to the experiment, all Beagle dogs underwent a health screening to exclude any cardiovascular or renal abnormalities. All dogs used in the study were obtained from a single supplier and were cared for by a dedicated staff at the animal facility. Each Beagle dog was assigned an identification number to ensure traceability. The dogs were housed individually in standard cages with free access to food and water, receiving a standard diet (250 ± 10 g/day). The animal care staff regularly monitored and recorded the vital signs, body weight, and behavioral status of each dog to ensure that the health and welfare of the animals met the experimental requirements. A total of 20 male Beagle dogs (approximately 5 kg each), induced with renal insufficiency via gentamicin administration, were prospectively included in the study. Using a random number generator, we assigned a random number to each beagle and then allocated them to one of the four experimental groups based on their assigned number. Each group consisted of five beagles, ensuring equal sample sizes between the experimental and control groups. The main ingredient of *Cordyceps sinensis* is fermented *Cordyceps sinensis* (*C. sinensis*, also known as Hirsutella sinensis) powder. *C. sinensis* belongs to the Clavicipitaceae family within the Ascomycota division. It is a parasitic fungus, rich in multiple active compounds, including cordycepin and its derivatives, polysaccharides, trace elements, and mycelia. The dosing regimen was 6 capsules per beagle three times a day. Group A received *Cordyceps sinensis* (produced by Hangzhou Zhongmei Huadong Pharmaceutical Co., Ltd., specification: 0.5 g per capsule, batch number: Z10910036) for 1 week, followed by manual low-dose CM CCTA scanning. Group B received *Cordyceps sinensis* for 1 week, followed by conventional dose CM CCTA scanning. Group C did not receive *Cordyceps sinensis* and underwent manual low-dose CM CCTA scanning after 1 week. Group D did not receive *Cordyceps sinensis* and underwent conventional dose CM CCTA scanning after 1 week. The study design is summarized in Table 1. All dogs were fasted prior to undergoing CCTA. The CM dosage was determined according to commonly used animal-equivalent dosage conversion tables. For adults, the standard dosage per kilogram of body weight was used as a reference, with an equivalent conversion factor of 1.5 for adult Beagle dogs, relative to humans. Based on the average adult human weight of 75 kg, the standard CM doses used for humans were 20, 30, 40, 50, and 60 mL. Corresponding dosages for Beagle dogs were 4, 6, 8, 10, and 12 mL, respectively. The conventional dose group received 12 and 14 mL, while the low-dose CM group received 4, 6, 8, and 10 mL. In this experiment, the experimental and control groups received 12 mL and 6 mL of CM for conventional and low-dose groups, respectively.

**TABLE 1:** Baseline data for Beagles.

Group		Cases	Gender	Intervention medications	Scanning type
Treatment group	Group A	5	M	*Cordyceps sinensis*	low dose contrast agent
	Group B	5	M	*Cordyceps sinensis*	Conventional dose contrast agent
Control group	Group C	5	M	Placebo	low dose contrast agent
	Group D	5	M	Placebo	Conventional dose contrast agent

### 2.2 Computed tomography examination

A CT scanner (uCT 960+, United Imaging Healthcare) was used to examine Beagle dogs. Prospective ECG gating was used. Scan parameters were X-ray tube rotation speed of 0.25s/r, tube voltage kV assist of 80–140 kV, baseline of 100kV, tube current Smart mA of 150–650 mA, and NI of 20. The detector covered 16 cm, KARL 3D, with a conventional scanning thickness of 0.50 mm. The number of scanning cycles is 1, Cardiac blood vessels related scanning parameters were: window width 800 HU and window location 180 HU. CTA data were transferred to CCTA raw images processed using automatic analysis software (SK-Coronary Doc, version 1.0, Shukun Technology, Beijing, China). The software algorithm used convolutional neural network to segment the aorta and coronary artery, extracted and named the center line from the segmentation skeleton, and generated the coronary artery tree structure using the minimum spanning tree to obtain the reconstructed image that met the requirements of coronary diagnosis. The software algorithm uses convolutional neural networks to segment the ventricle, myocardium, aorta, and coronary arteries to obtain a reconstructed image that meets the requirements of coronary diagnosis.

### 2.3 Computed tomography scanning protocols

#### 2.3.1 Protocol for conventional dose CM group

12 mL of non-ionic CM iomeprol (400 mg/mL) was injected through the forelimb vein with a high-pressure syringe at a flow rate of 4–5 mL/s. The scanning range was the whole heart. The threshold of the ascending aorta was set to 80 HU using the conventional automatic trigger protocol. All CTA scans were performed in a single heartbeat.

#### 2.3.2 Protocol for low dose CM group

The monitoring level was placed in the ascending aorta, and 6 mL of CM iomeprol (400 mg/mL) was injected at a flow rate of 4–5 mL/s during scanning. The scan time was set according to beagle’s heart rate (HR): HR > 100 bmp, scanning trigger timing: 6–7 s after the injection of CM and initiated manually with a delay of 2s; HR = 90-100 bmp, scanning trigger timing: 8–9 s after the injection of CM and initiated manually with a delay of 2 s. HR < 90 bmp, scanning trigger timing: 10–11 s after the injection of CM and initiated manually with a delay of 2 s.

### 2.4 Renal MRI scanning sequences and parameters

After establishing the Beagle dog model of renal insufficiency, 3.0T BOLD-MRI imaging was performed using a MAGNETOM Vida (Siemens Healthineers, Germany). Prior to scanning, both the experimental and control groups were fasted for 6 h to avoid food-induced motion artifacts caused by gastric peristalsis. Five minutes before the scan, anesthesia was administered by intravenous injection of a specialized animal experimental formulation, Sufentanil 50 (fentanyl 125 mg + zolazepam 125 mg) and Selazepam, mixed with physiological saline at a 10:1:89 ratio. The mixture was injected intravenously in the forelimb at a dose of 2.0 mL/kg, based on the body weight of each Beagle dog. A 14-channel abdominal coil was used, with the dogs positioned in a prone, head-first orientation. The scanning region was centered on the kidney area, covering the entire kidney. The abdomen was secured with a belt, and sandbags were placed in the center of the coil to minimize respiratory motion artifacts. During scanning, saturation bands were applied to suppress the effects of intestinal air. Additionally, the BLADE technique was employed in the T2-weighted imaging (T2WI) sequence to eliminate motion artifacts. BOLD imaging was performed using a multi-gradient echo sequence with 8 slices, a slice thickness of 5 mm, a field of view (FOV) of 204 × 204 mm, 4 excitations, TR = 395 ms, and TE = 26. IVIM imaging was carried out using a single spin-echo diffusion-weighted imaging sequence, with b-values set at 0, 30, 50, 100, 200, 400, 600, 800, and 1,200 s/mm^2^. The sequence consisted of 8 slices with a slice thickness of 3.5 mm, FOV of 204 × 204 mm, 1 excitation, TR = 2,600 ms, and TE = 97 ms.

### 2.5 MRI data analysis

The D, D*, f and R2* values of renal cortex and medulla were measured. The conventional dose and low dose contrast medium coronary CT angiography were compared and analyzed. The specific steps are as follows: The raw data of IVIM and BOLD of coronary CT angiography of beagle dogs with and without *Cordyceps sinensis* were uploaded to Siemens Syngo. via post-processing workstation, respectively. Two radiologists with senior professional titles processed the data in a blind way to obtain the D, D*, f and R2* pseudo-color maps. The ROI was delineated at the level proximal to the renal hilum of the original IVIM image, avoiding blood vessels, renal pelvis, and renal margins. The renal cortex ROI was an arc covering 60–80 mm^2^ of the current slice of the cortex. The renal medulla ROI was a 20 mm^2^ circular region in the upper, middle and lower pole of the kidney. All rois were automatically matched to the D, D*, f and R2* pseudo-color images, and the corresponding parameters of the right kidney cortex and medulla were measured, and the mean value at each time point was used as the final result. All beagle dogs underwent renal functional MRI before and after coronary CT angiography with conventional and low dose contrast medium. Intravoxel incoherent motion (IVIM) and blood oxygen level dependent (BOLD) images of the kidney were obtained. The key MRI parameters such as pure diffusion coefficient (D), pseudo-diffusion coefficient (D*), perfusion fraction (f) and R2* value were measured. Two physicians independently measured IVIM and BOLD parameters in a blinded manner, in order to evaluate the inter-observer agreement, two radiologists performed the same MRI images with two repeated measurements independently, and the average values of each radiologist were compared to evaluate the differences between the measurements.

### 2.6 Image evaluation

Subjective image quality evaluation: two independent cardiovascular radiologists with 30 years of experience in cardiovascular radiology and scored CCTA coronary images using a dedicated image workstation (Syngo.via, version 60, Siemens Healthcare, Germany). During the evaluation, CCTA images were randomly selected and evaluated by radiologists who were blinded to image reconstruction and experimental grouping. In cases of disagreement, a final score was reached by consensus among the physicians. Subjective image quality was scored using a 5-point scale (1 = unassessble, 5 = excellent): 1, does not meet the diagnosis; 2, poor image quality; 3, basically in line with the diagnosis; 4, the image quality is good; 5, the image quality is high. The radiation dose and CM dosage were also recorded.

Objective image quality evaluation:Measurements were made in the proximal segment of the main coronary artery, right coronary artery (RCA), left circumflex branch (LCX), left anterior descending branch (LAD), aorta and para-aortic myocardial tissue. Coronary ROIs had a diameter of 1.5 mm.

### 2.7 Renal function parameters measurement

Blood samples were collected from Beagle dogs before and after CCTA imaging with both conventional and low-dose CM. Serum creatinine (Scr), blood urea nitrogen (BUN), cystatin C (Cys C), neutrophil gelatinase-associated lipocalin (NGAL), and the albumin-to-globulin ratio were measured using a biochemical analyzer. Changes in these renal function indicators before and after the experiment were analyzed. Additionally, the correlations between laboratory parameters and MRI parameters were evaluated.

### 2.8 Statistical analysis

All data analyses were performed using SPSS software (version 25.0, IBM, Armonk, NY, United States), and scatter plots were created using GraphPad Prism (version 9.5.0,GraphPad Software, San Diego, CA, United States) statistical software,and post Power analysis was performed using G*Power software. The normality of all continuous variables was tested. Variables with a normal distribution were described as mean ± standard deviation (Mean ± SD); variables not following a normal distribution were described using the median and interquartile range (IQR). The Kappa and intraclass correlation coefficients (ICC) were used to detect the consistency of the assessments among different radiologists. The rank sum test was applied to compare score differences between groups. Paired sample t-tests or Wilcoxon signed-rank tests were used to evaluate the impact of *Cordyceps sinensis* on renal function indicators in the experimental and control groups. Within-group comparisons were performed using one-way analysis of variance (ANOVA). Pearson correlation analysis was used to examine the correlation between renal MRI parameters and laboratory indicators, with 0.40 ≤ |r| ≤ 0.69 indicating moderate correlation and 0.70 ≤ |r| ≤ 0.89 indicating strong correlation. All statistical tests were two-sided, and a P-value of <0.05 was considered statistically significant.

## 3 Results

### 3.1 Image quality and CT values

**FIGURE 1:**
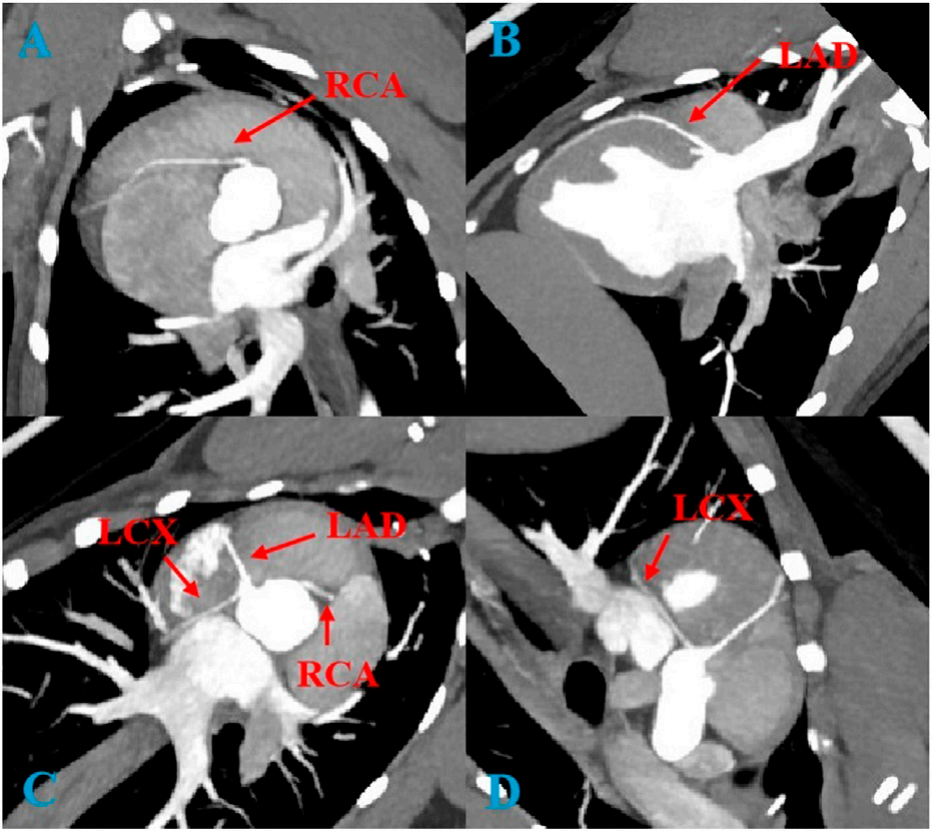
Coronary CTA images of a Beagle receiving 6 ml of contrast agent. **(A)**: RCA, right coronary artery; **(B)**: LAD, left anterior descending artery; **(C)**: Three main coronary arteries; **(D)**: LCX, left circumflex artery.

Figure 1 is a representative image of coronary artery CTA. In the imaging analysis, the CT values (expressed as mean ± standard deviation) of the left anterior descending artery (LAD), right coronary artery (RCA), and left circumflex artery (LCX) in both the experimental and control groups were consistent with the international diagnostic standards for coronary arteries (CT values >250 HU). This demonstrated that both conventional and low-dose CCTA scans provided high-quality images. Image quality analysis revealed no significant differences between the experimental and control groups. These results are presented in Tables 2, 3.

**TABLE 2:** Comparison of subjective evaluation of CCTA image quality.

Group	Cases	Image quality (points)				
		1 (cases, %)	2 (cases, %)	3 (cases, %)	4 (cases, %)	5 (cases, %)
Group A	5	0 (0)	0 (0)	1 (20)	2 (40)	2 (40)
Group B	5	0 (0)	0 (0)	0 (0)	3 (60)	2 (40)
Group C	5	0 (0)	0 (0)	0 (0)	4 (80)	1 (20)
Group D	5	0 (0)	0 (0)	1 (20)	3 (60)	1 (20)
H		0.98				
*P*		0.806				

**TABLE 3:** Comparison of objective evaluation of CCTA image quality.

Group	Cases	LAD CT values ( $\bar{x}$ ±s)	LCX CT values ( $\bar{x}$ ±s)	RCA CT values ( $\bar{x}$ ±s)
Group A (6 mL)	5	353.7 ± 17.4	357.1 ± 16.5	357.4 ± 15.9
Group B (12 mL)	5	365.1 ± 11.4	375.3 ± 11.5	375.3 ± 14.3
Group C (6 mL)	5	354.1 ± 15.3	355.7 ± 15.9	355.5 ± 15.4
Group D (12 mL)	5	369.4 ± 19.5	375.5 ± 14.7	370.1 ± 20.2
F		1.198	2.777	1.693
*P*		0.342	0.075	0.209

### 3.2 IVIM and BOLD MRI parameter comparison analysis

**FIGURE 2:**
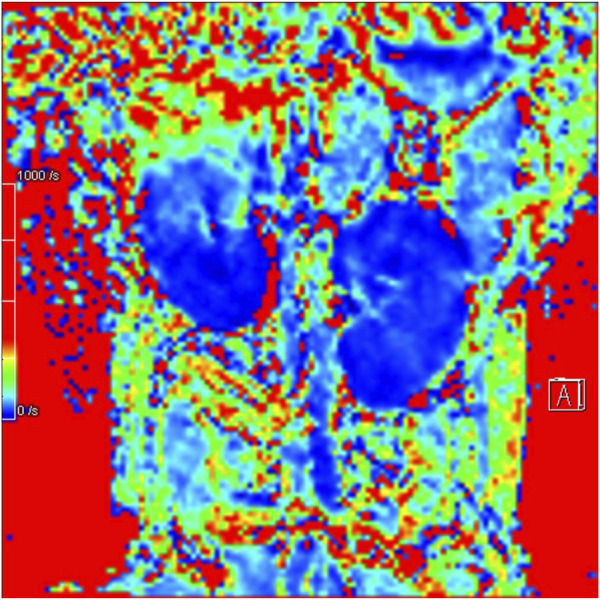
Representative BOLD image from the experimental group.

**FIGURE 3:**
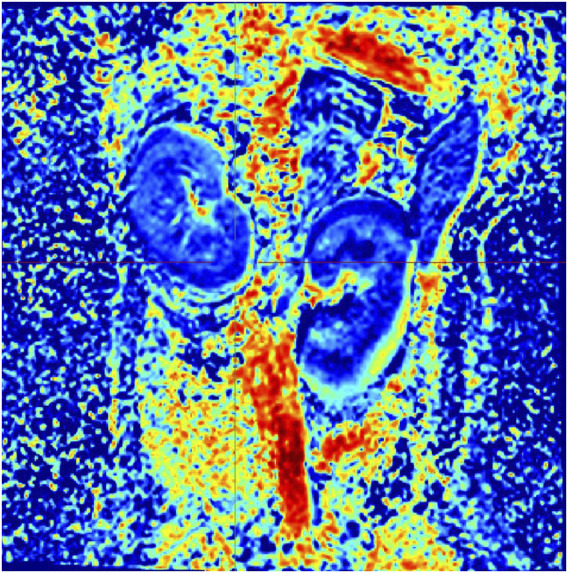
Representative IVIM image from the experimental group.

In Group A, Beagle dogs that were administered *Cordyceps sinensis* for 7 days and underwent low-dose CM coronary CT imaging showed an increase in IVIM parameters (including D, D*, and f values in both the renal cortex and medulla) and a decrease in BOLD parameters. Figures 2, 3 show the representative BOLD and IVIM images of the experimental group. In Group B, Beagle dogs that were administered *Cordyceps sinensis* for 7 days and underwent conventional dose CM coronary CT imaging showed no significant changes in the IVIM and BOLD parameters of the kidneys. In Group C, Beagle dogs that were not administered *Cordyceps sinensis* and underwent low-dose CM coronary CT imaging also showed no significant changes in the IVIM and BOLD parameters. In Group D, Beagle dogs that were not administered *Cordyceps sinensis* and underwent conventional dose CM coronary CT imaging exhibited a significant decrease in IVIM parameters and a significant increase in BOLD parameters. When comparing the IVIM and BOLD parameters of the kidneys after coronary CT imaging across the four groups, significant differences were found in the D, D*, f, and R2* values between Groups A and B, as well as between Groups C and D (P < 0.05). These results suggest that low-dose CM coronary CT imaging provides a degree of renal protection in Beagle dogs. Furthermore, significant differences in the D, D*, f, and R2* values in the renal cortex and medulla were observed between Groups A and C, and between Groups B and D (P < 0.05), indicating that *Cordyceps sinensis* administration offers renal protection in Beagle dogs. These findings are summarized in Table 4. The intra-group consistency test of the measurement results of doctors A and B is shown in Table 5. A *post hoc* power analysis was conducted. Based on a medium effect size f = 0.25 and a four-group one-way ANOVA design, the study had a power of 0.91 (91%) at the set significance level α = 0.05.

**TABLE 4:** Comparison of kidney IVIM and BOLD parameters.

Group		Cortex				Medulla			
		D value (×10^−4^ mm^2^/s)	D^*^value (×10^−3^ mm^2^/s)	f value (%)	R2^*^value	D value (×10^−4^ mm^2^/s)	D^*^value (×10^−3^ mm^2^/s)	f value (%)	R2^*^value
Group A	Change	4.34 ± 3.18	17.06 ± 10.57	20.28 ± 16.27	7.60 ± 2.68	9.44 ± 3.03	16.10 ± 7.48	12.00 ± 5.05	9.68 ± 3.78
	t	−3.049	−3.608	−2.788	6.347	−6.969	−4.814	−5.318	5.739
	*P*	0.038^*^	0.023^*^	0.049^*^	0.003^*^	0.002^*^	0.009^*^	0.006^*^	0.005^*^
Group B	Change	4.66 ± 4.63	7.94 ± 13.85	3.64 ± 18.83	2.78 ± 3.36	1.60 ± 3.46	7.70 ± 16.13	1.22 ± 13.97	1.12 ± 1.95
	t	2.251	−1.282	0.432	−1.852	−1.033	1.067	−0.195	−1.284
	*P*	0.088	0.269	0.688	0.138	0.360	0.346	0.855	0.269
Group C	Change	1.54 ± 2.45	5.60 ± 8.88	11.44 ± 22.91	0.12 ± 1.26	2.94 ± 3.38	3.08 ± 11.95	15.80 ± 16.40	0.08 ± 2.95
	t	1.404	1.41	1.116	−0.213	−1.947	0.576	2.155	−0.061
	*P*	0.233	0.231	0.327	0.842	0.123	0.595	0.097	0.954
Group D	Change	7.58 ± 3.24	18.18 ± 8.86	31.46 ± 24.78	6.68 ± 0.93	8.14 ± 3.50	19.26 ± 7.25	20.94 ± 13.50	9.46 ± 2.52
	t	5.238	4.587	2.84	−16.042	5.204	5.942	3.469	−8.406
	*P*	0.006^*^	0.01^*^	0.047^*^	<0.001^*^	0.006^*^	0.004^*^	0.026^*^	0.001^*^

**TABLE 5:** Analysis of the consistency in test results of two physicians.

Parameters	Cortex				Medulla			
	D value (×10^−4^ mm^2^/s)	D^*^value (×10^−3^ mm^2^/s)	f value (%)	R2^*^value	D value (×10^−4^ mm^2^/s)	D^*^value (×10^−3^ mm^2^/s)	f value (%)	R2^*^value
ICC	0.819	0.756	0.857	0.764	0.792	0.843	0.818	0.747
P	<0.0001*	<0.0001*	<0.0001*	<0.0001*	<0.0001*	<0.0001*	<0.0001*	<0.0001*

### 3.3 Changes in laboratory renal function indicators

In group A, the laboratory renal function indexes decreased before and after the experiment, but there was no significant change in group B, indicating that *Cordyceps sinensis* has a certain protective effect on renal function. There were no significant changes in the renal function indexes in group C before and after the experiment, while the renal function indexes in group D increased, indicating that the CM aggravated the renal function injury in the control beagle dogs (Table 6). There were significant differences in serum creatinine, urea nitrogen, cystatin C and NGAL between groups A and B and groups C and D (P < 0.05), indicating that low-dose CM coronary CT angiography had a certain protective effect on the kidneys of beagle dogs. There were significant differences in serum creatinine, urea nitrogen, cystatin C and NGAL between groups A and C and groups B and D (P < 0.05), which indicated that taking Cordyceps sinicus had certain protective effect on Beagle’s kidney (Table 7).

**TABLE 6:** Comparison of laboratory indexes.

Group		Scr	BUN	Cys C	NGAL	A/G
Group A	Change	91.22 ± 17.05	12.67 ± 0.77	0.14 ± 0.02	2.25 ± 0.73	0.13 ± 0.13
	t	11.961	37.042	13.924	6.929	−2.246
	*P*	<0.001^*^	<0.001^*^	<0.001^*^	0.002^*^	0.088
Group B	Change	5.34 ± 29.22	0.82 ± 1.34	0.02 ± 0.09	0.21 ± 0.39	0.11 ± 0.10
	t	−0.409	−1.364	−0.614	−1.209	−2.31
	*P*	0.704	0.244	0.572	0.293	0.082
Group C	Change	12.82 ± 31.20	0.18 ± 1.24	0.04 ± 0.04	0.09 ± 0.35	0.08 ± 0.10
	t	−0.919	0.324	−2.53	0.58	−1.802
	*P*	0.41	0.762	0.065	0.593	0.146
Group D	Change	90.22 ± 17.82	13.12 ± 5.35	0.92 ± 0.31	1.94 ± 1.67	0.084 ± 0.21
	t	−11.32	−5.486	−6.68	−5.608	0.904
	*P*	<0.001^*^	0.005^*^	0.003^*^	0.006^*^	0.417

**TABLE 7:** Group comparisons of renal fMRI parameters and laboratory indexes.

Parameters		Group A	Group B	Group C	Group D	F	*P*
Laboratory indexes	Scr	243.04 ± 11.71	352.36 ± 8.37*a*	344.86 ± 19.16	408.14 ± 51.82	29.020	<0.0001^*^
	BUN	20.87 ± 0.90	32.46 ± 1.45*a*	31.10 ± 1.43	46.70 ± 6.87	43.330	<0.0001^*^
	Cys C	0.40 ± 0.08	0.71 ± 0.04*a*	0.61 ± 0.13	1.55 ± 0.17	95.574	<0.0001^*^
	NGAL	1.69 ± 0.16	3.88 ± 0.36*a*	3.83 ± 0.53	6.17 ± 1.06	42.694	<0.0001^*^
Cortex	R2^*^value	34.70 ± 2.87	40.82 ± 4.78*a*	38.42 ± 1.99	48.22 ± 2.82	15.162	<0.0001^*^
	D value (×10^−4^ mm^2^/s)	20.02 ± 4.70	11.92 ± 3.27*a*	15.36 ± 3.96	7.14 ± 1.96	9.821	0.001^*^
	D^*^value (×10^−3^ mm^2^/s)	70.40 ± 4.31	53.34 ± 6.48*a*	50.86 ± 9.18	33.86 ± 5.85	24.986	<0.0001^*^
	f value (%)	135.24 ± 7.64	97.28 ± 8.98*a*	95.94 ± 9.45	79.42 ± 10.56	32.834	<0.0001^*^
Medulla	R2^*^value	27.78 ± 3.50	38.70 ± 1.47*a*	36.08 ± 2.52	46.78 ± 3.39	38.000	<0.0001^*^
	D value (×10^−4^ mm^2^/s)	22.88 ± 4.23	13.28 ± 4.52*a*	15.10 ± 4.24	6.22 ± 2.33	15.184	<0.0001^*^
	D^*^value (×10^−3^ mm^2^/s)	60.40 ± 4.60	41.70 ± 8.78*a*	40.18 ± 6.98	27.88 ± 8.76	16.091	<0.0001^*^
	f value (%)	111.64 ± 7.76	84.96 ± 7.49*a*	83.50 ± 6.37	68.66 ± 7.54	29.942	<0.0001^*^

Note. *a* represents comparison with group C, P > 0.05. All pairwise comparisons were corrected by Bonferroni.

### 3.4 Correlation between renal fMRI and laboratory indicators

**FIGURE 4:**
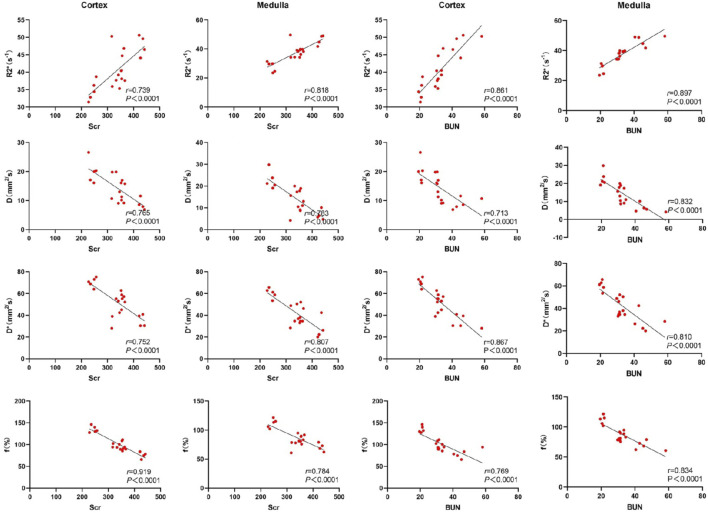
Scatter chart of correlation between renal fMRI and Scr and BUN.

**FIGURE 5:**
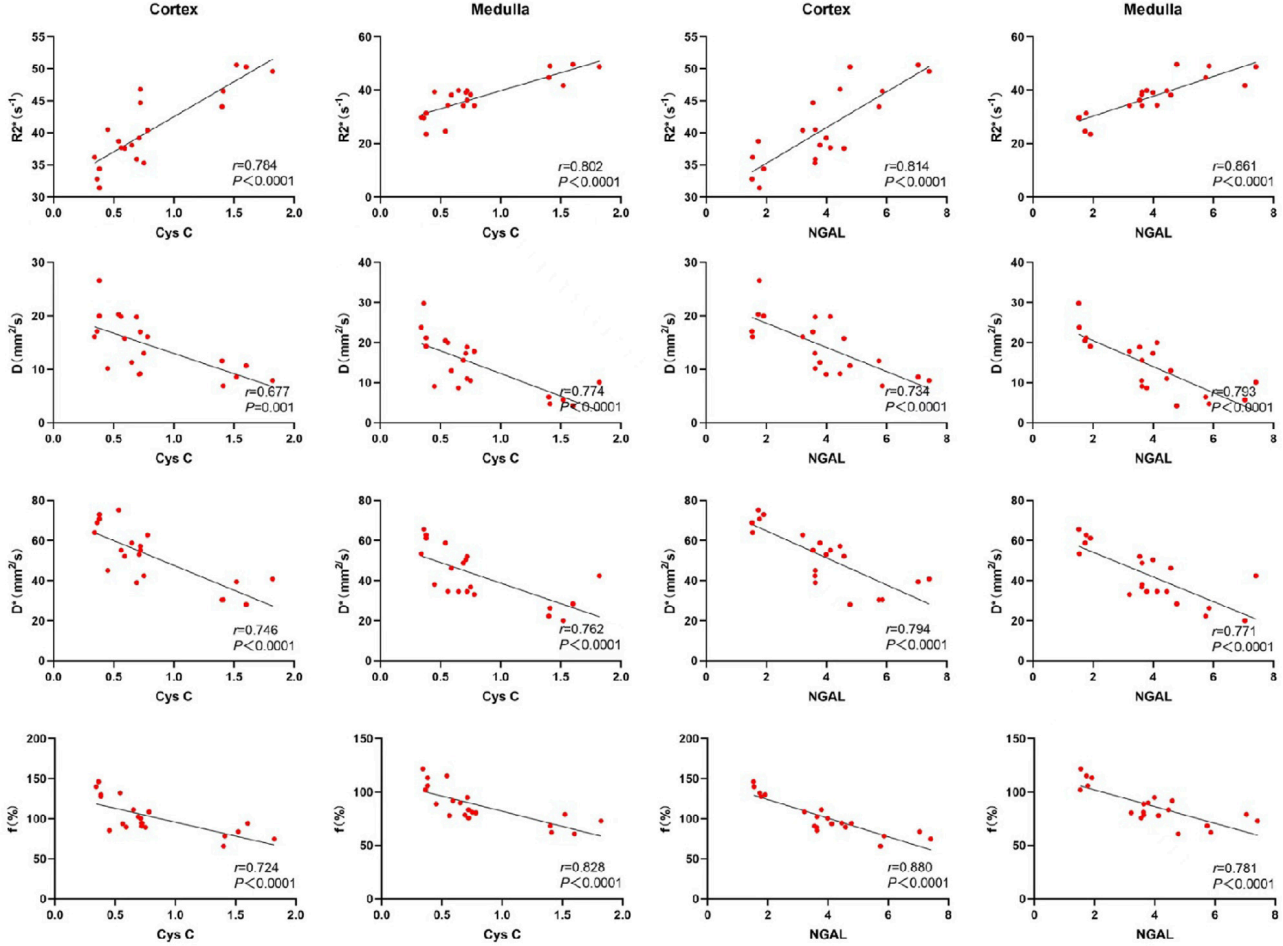
Scatter chart of correlation between renal fMRI and Cys C and NGAL.

Serum creatinine, blood urea nitrogen (BUN), and neutrophil gelatinase-associated lipocalin (NGAL) were highly correlated with the R2* values, D, D*, and f values in both the renal cortex and medulla. Cystatin C exhibited a moderate correlation with the D values in the renal cortex, and a strong correlation with R2* values, D*, and f values in the renal cortex. Additionally, cystatin C was strongly correlated with R2* values, D, D*, and f values in the renal medulla. These results indicate that key functional MRI parameters can non-invasively reflect renal function status. See Figures 4, 5.

## 4 Discussion and conclusion

The results of this study indicate that low-dose CM coronary CT angiography (CCTA) is still capable of obtaining high-quality images in a Beagle dog model of renal insufficiency. In terms of diagnostic performance and image quality, we reduced the standard CM dose for Beagle dogs by 50% (from 12 mL to 6 mL), and found no significant differences in the image quality of the three main coronary arteries between the two dosing schemes. Currently, the Society of Cardiovascular CT guidelines recommend a minimum vascular attenuation of 250 HU for coronary CTA (Abbara et al., 2016). Becker et al. (2003) confirmed that coronary artery enhancement values of approximately 250–300 HU meet diagnostic standards, aiding in the identification of calcified plaques in the coronary artery walls. In our study, the low-dose CM and heart rate-based scanning time were similar to the range reported by the Society of Cardiovascular CT and Becker et al., suggesting that the use of a smaller CM dose (12 mL) in this study is beneficial as it reduces the potential risk of exacerbating renal insufficiency in Beagle dogs.

*Cordyceps sinensis* has been shown to possess various therapeutic effects, including blood sugar regulation, anti-inflammatory properties, immune modulation, antioxidant activity, and antifibrotic effects. It has been widely used in clinical practice to treat respiratory diseases, immune disorders, kidney diseases, and even various types of cancer (Zhang et al., 2014; Tan et al., 2022). This study demonstrates that *Cordyceps sinensis* may offer protective effects against contrast-induced renal injury. In Group A, Beagle dogs administered *Cordyceps sinensis* showed increased IVIM (D, D*, and f values in both the renal cortex and medulla) and decreased BOLD parameters (R2* values), along with improved renal function indicators. In contrast, Group B, also administered *Cordyceps sinensis*, showed no significant changes in renal function, IVIM, or BOLD parameters. In Group C, Beagle dogs that did not receive *Cordyceps sinensis* showed no significant changes in renal function indicators, IVIM, or BOLD parameters. However, in Group D, Beagle dogs that did not receive *Cordyceps sinensis* exhibited a significant decrease in IVIM parameters and an increase in BOLD parameters, along with worsened renal function indicators. A comparative analysis of the four groups revealed significant differences in the D, D*, f values, R2* values, serum creatinine, BUN, cystatin C, and NGAL between Groups A and C, and Groups B and D (P < 0.05). This suggests that both low-dose CM CCTA and *Cordyceps sinensis* administration provide protective effects on renal function in Beagle dogs, which is of significant clinical relevance for coronary CT imaging in patients with renal insufficiency. These findings align with previous studies (Liang et al., 2016; Tao et al., 2024b; Liu et al., 2022; Zhang et al., 2014; Zhao et al., 2022). Furthermore, significant differences in the same parameters were observed between Groups A and B, and Groups C and D, indicating that *Cordyceps sinensis* may have a protective role in renal function. The results suggest that lowering the CM dose or using renal protective agents, such as *Cordyceps sinensis*, can improve the safety of CCTA in special pathological conditions.

An increase in R2* values indicates that CM injection can cause local microenvironmental obstruction in the kidneys, reducing tissue perfusion and altering the magnetic susceptibility of local voxels, which results in an increase in deoxygenated hemoglobin, a marker of kidney hypoxia. In this study, we used multi-parameter functional MRI to monitor early changes in renal oxygen metabolism following iodine contrast injection through BOLD imaging, and to assess blood flow and water molecule movement changes via IVIM, offering a comprehensive and accurate view of renal function changes after contrast injection. Prasad et al. (2023) demonstrated the feasibility of using BOLD imaging for non-invasive quantitative evaluation of renal oxygen content in humans, and Liang et al. (2016) used IVIM to study the pathophysiological processes of contrast-induced acute kidney injury in rats. These findings suggest that functional MRI renal imaging can detect renal pathophysiological changes, such as diffusion and perfusion injury, reduced oxygen consumption, and increased oxygen concentration.

A strong correlation was observed between functional MRI renal parameters and laboratory indicators in this study. Specifically, serum creatinine, cystatin C, BUN, and NGAL levels were positively correlated with R2* values and negatively correlated with D, D*, and f values in both the renal cortex and medulla. These results are consistent with previous studies (Mao et al., 2023; Chen et al., 2021; Copur et al., 2022), and indicate that functional MRI renal parameters are important indicators of the severity of chronic kidney damage (Li et al., 2019). Functional MRI can serve as an effective and non-invasive tool for detecting renal function.

The limitations of this study include its single-center design and the relatively small sample size. Future studies should aim to expand the sample size to further validate the renal protective effects of *Cordyceps sinensis* at different CM doses and assess its potential for use in other imaging modalities. The treatment duration and follow-up period after contrast administration were not involved in this study, which is what we will do in the future.

Coronary CT angiography (CCTA) can achieve high-quality imaging in a Beagle dog model of renal insufficiency. *Cordyceps sinensis* may have a protective effect on renal function, providing new insights and references for the clinical application of CCTA in patients with renal insufficiency. The combination of IVIM and BOLD functional MRI offers a non-invasive method to assess the renal protective effects of *Cordyceps sinensis* in Beagle dogs with renal insufficiency before and after CCTA. The approach is safe, feasible, and exhibits high reproducibility and repeatability, making it a promising tool for clinical application.

## Data Availability

The original contributions presented in the study are included in the article/supplementary material, further inquiries can be directed to the corresponding author.

## Abbreviations

CM, contrast medium; CCTA, coronary; CT, angiography; LAD, left anterior descending artery; LCX left, circumflex artery; RCA, right coronary artery.
